# Inhibition of MER proto-oncogene tyrosine kinase by an antisense oligonucleotide enhances treatment efficacy of immunoradiotherapy

**DOI:** 10.1186/s13046-024-02992-2

**Published:** 2024-03-06

**Authors:** Yun Hu, Alexey Revenko, Hampartsoum Barsoumian, Genevieve Bertolet, Natalie Wall Fowlkes, Hadi Maazi, Morgan Maureen Green, Kewen He, Duygu Sezen, Tiffany A. Voss, Claudia S Kettlun Leyton, Fatemeh Masrorpour, Zahid Rafiq, Nahum Puebla-Osorio, Carola Leuschner, Robert MacLeod, Maria Angelica Cortez, James W. Welsh

**Affiliations:** 1 Department of Radiation Oncology, The University of Texas MD Anderson Cancer Center, Houston, Texas USA; 2 Ionis Pharmaceuticals, Carlsbad, CA 92008 USA; 3 Department of Veterinary Medicine & Surgery, The University of Texas MD Anderson Cancer Center, Houston, Texas USA; 4 Department of Radiation Oncology, Shandong Cancer Hospital and Institute, Shandong First Medical University and Shandong Academy of Medical Sciences, Jinan, China; 5 Department of Radiation Oncology, Koc University School of Medicine, Istanbul, Turkey

**Keywords:** Immunoradiotherapy, Immunotherapy resistance, MerTK, Antisense oligonucleotide, M1 macrophage, Antitumor immune response

## Abstract

**Background:**

The combination of radiotherapy and immunotherapy (immunoradiotherapy) has been increasingly used for treating a wide range of cancers. However, some tumors are resistant to immunoradiotherapy. We have previously shown that MER proto-oncogene tyrosine kinase (MerTK) expressed on macrophages mediates resistance to immunoradiotherapy. We therefore sought to develop therapeutics that can mitigate the negative impact of MerTK. We designed and developed a MerTK specific antisense oligonucleotide (ASO) and characterized its effects on eliciting an anti-tumor immune response in mice.

**Methods:**

344SQR cells were injected into the right legs on day 0 and the left legs on day 4 of 8-12 weeks old female 129sv/ev mice to establish primary and secondary tumors, respectively. Radiation at a dose of 12 Gy was given to the primary tumors on days 8, 9, and 10. Mice received either anti-PD-1, anti-CTLA-4 or/and MerTK ASO starting from day 1 post tumor implantation. The composition of the tumor microenvironment and the level of MerTK on macrophages in the tumor were evaluted by flow cytometry. The expression of immune-related genes was investigated with NanoString. Lastly, the impact of MerTK ASO on the structure of the eye was histologically evaluated.

**Results:**

Remarkably, the addition of MerTK ASO to XRT+anti-PD1 and XRT+anti-CTLA4 profoundly slowed the growth of both primary and secondary tumors and significantly extended survival. The ASO significantly reduced the expression of MerTK in tumor-associated macrophages (TAMs), reprograming their phenotype from M2 to M1. In addition, MerTK ASO increased the percentage of Granzyme B^+^ CD8^+^ T cells in the secondary tumors when combined with XRT+anti-CTLA4. NanoString results demonstrated that the MerTK ASO favorably modulated immune-related genes for promoting antitumor immune response in secondary tumors. Importantly, histological analysis of eye tissues demonstrated that unlike small molecules, the MerTK ASO did not produce any detectable pathology in the eyes.

**Conclusions:**

The MerTK ASO can significantly downregulate the expression of MerTK on TAMs, thereby promoting antitumor immune response. The combination of MerTK ASO with immunoradiotherapy can safely and significantly slow tumor growth and improve survival.

**Graphical Abstract:**

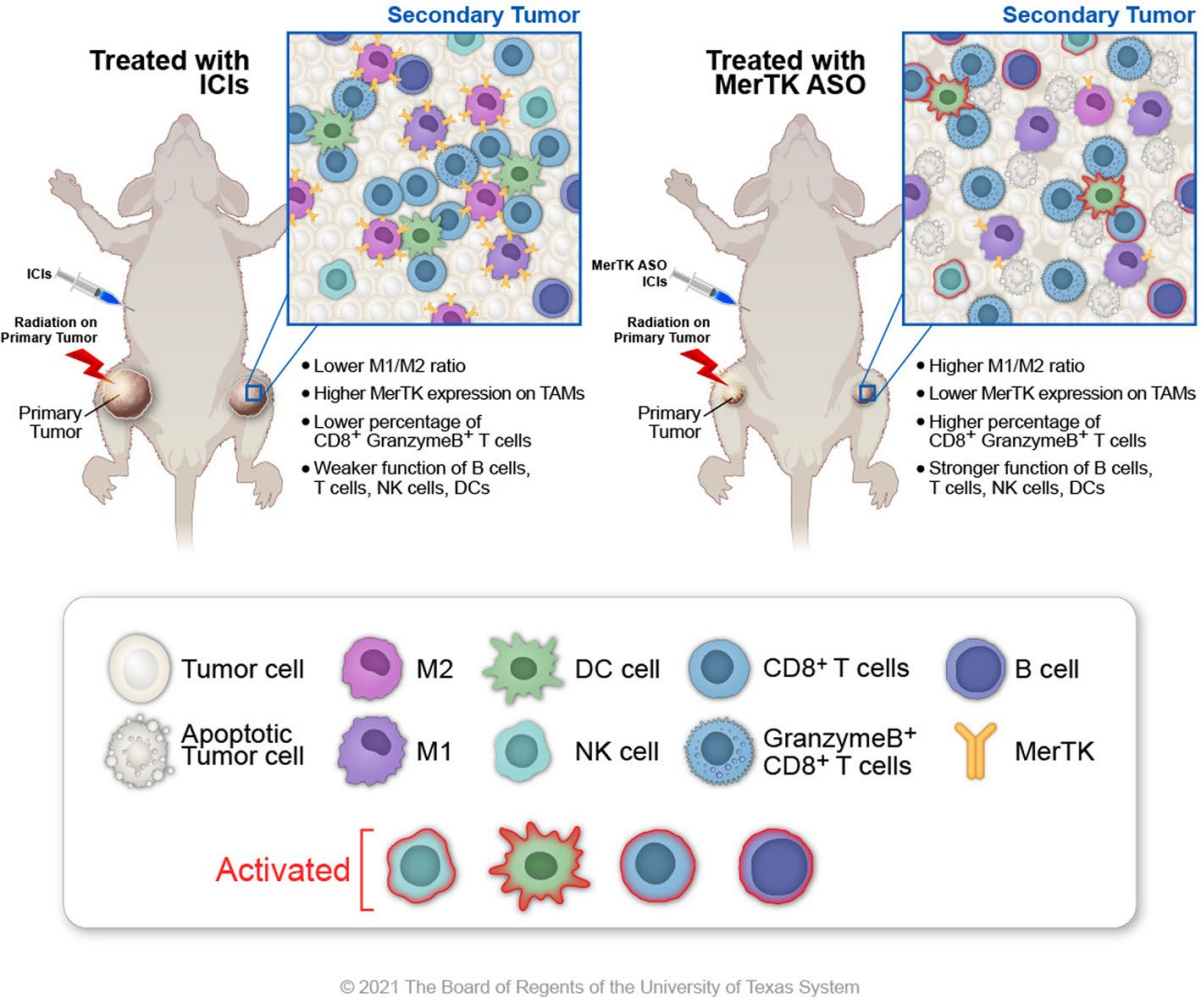

**Supplementary Information:**

The online version contains supplementary material available at 10.1186/s13046-024-02992-2.

## Background

The combination of immunotherapy and radiotherapy (immunoradiotherapy) has been increasingly used in the treatment of various types of cancers in recent years [1–3]. Radiation can induce apoptosis in tumor cells and subsequently trigger the activation of tumor-specific immune responses, which, with the help from immune checkpoint blockade, can effectively eradicate both locally irradiated and remote cancer cells. One of the key factors hampering this process is premature clearance of apoptotic cancer cells by macrophages acting to prevent excessive inflammation [4, 5]. An undesirable consequence of this is inhibition of inflammatory responses and suppression of anti-tumor immunity [6].

A critical receptor that mediates the clearance of apoptotic cells by macrophages is MER proto-oncogene tyrosine kinase (MerTK). Together with TYRO3 and AXL, MerTK is a member of a receptor tyrosine kinase family [7]. MerTK is overexpressed or ectopically expressed in a wide variety of cancers, including leukemia, non–small cell lung cancer, and glioblastoma, and can potentially activate several canonical oncogenic signaling pathways [8]. One of the main mechanisms by which MerTK suppresses immune activation is through the prevention of intracellular content release from apoptotic cells by inducing efferocytosis [9]. In addition, MerTK signaling promotes the secretion of anti-inflammatory cytokines, such as transforming growth factor-β (TGFβ), hepatocyte growth factor (HGF), and IL-10, resulting in immunosuppressive, pro-tumor, M2-polarized macrophages in the tumor microenvironment (TME) [9, 10]. Thus, macrophage MerTK activity is an attractive therapeutic target for cancer treatment [10, 11].

Small molecules and monoclonal antibody inhibitors of MerTK have been identified [12, 13]. Cummings and colleagues reported that Mer590, a MerTK-targeting monoclonal antibody, reduced non-small cell lung cancer (NSCLC) colony formation *in vitro* [13]. *In vivo* studies also confirmed that MerTK blockade by monoclonal antibodies promoted antitumor immunity, and improved the control of tumors in various tumor models [6, 14, 15]. We have previously demonstrated that the combination of an anti-MerTK (αMerTK) antibody, αPD1, and radiotherapy increased the activated CD8^+^ and NK cell populations within the abscopal TME, leading to delayed abscopal tumor growth, improved survival rates, and reduced numbers of lung metastases in a murine metastatic adenocarcinoma NSCLC model [4]. UNC569, a small molecule MerTK inhibitor, also displayed potent antitumor efficacy both *in vitro* and *in vivo* [12, 16]. However, anti-MerTK antibodies and UNC569 have caused severe toxicity to the retina in preclinical models, precluding any likely clinical applications [17, 18]. This is likely due to the blocking effects of the antibody and UNC569 on MerTK in the eyes, where it plays an essential homeostatic role in the retina [19]. Therefore, it is necessary to develop novel therapeutics that efficiently block pro-tumor MerTK in the periphery while sparing MerTK in the eyes.

To this end, we designed a MerTK specific antisense oligonucleotide (ASO) and tested its efficacy and safety in mice. ASOs are small, synthetic, single-stranded nucleic acid polymers, which can be employed to modulate gene expression *via* various mechanisms [20]. Due to their high specificity, good safety profile, and potent efficacy, ASOs are considered promising therapies for a wide range of diseases [21, 22]. We found that the combination of MerTK ASO with radiotherapy and anti-PD1 or anti-CTLA4 significantly enhances the anti-tumor immune response and improves treatment efficacy in both the primay, irradiated tumors and secondary, unirradiated tumors in an anti-PD1-resistant lung cancer model. Additionally, we discovered that MerTK ASO, in conjunction with chemotherapy, MEK inhibitor (AZD6244) and anti-PDL1, and anti-PD1 improves tumor control in various tumor models. Importantly, we did not observe any pathological effects in the eyes after treatment with MerTK ASO.

## Materials and methods

### Cell line

344SQR, an αPD1 resistant lung cancer cell line, was generated in a previous study and was used throughout this study [23]. The cell line was cultured in complete medium (RPMI-1640 supplemented with 100 units/mL penicillin, 100 μg/mL streptomycin, and 10% heat-inactivated fetal bovine serum) and incubated at 37 °C in 5% CO_2_.

### Materials

A mouse MerTK-specific ASO and control ASOs were produced by Ionis Pharmaceuticals, Inc. Anti-mouse CTLA-4 (Catalog# BP0164) and anti-mouse PD-1 (Catalog# BE0146) were purchased from BioXCell. Liberase (Catalog #05401127001) and DNAse (Catalog #4716728001) were purchased from Roche and Sigma-Aldrich, respectively. Flow cytometry antibodies, including αCD45-PerCP Cy5.5 (Catalog# 103131), αCD4-PE/Dazzle594 (Catalog# 100456), αCD8-FITC (Catalog# 100706), αGranzyme B (GrB)-Pacific Blue (Catalog# 515408), αGr1-BV510 (Catalog# 108437), αCD11b-APC Fire750 (Catalog# 101262), αF4/80-Alexa Fluor 700 (Catalog# 123130), αCD38-PE-Cy7 (Catalog# 102718), αCD206-PE (Catalog# 141706), and αMertK-APC (Catalog# 151507) were ordered from BioLegend.

### Tumor inoculation and treatment

5x10^4^ 344SQR cells in 100 μL phosphate-buffered saline (PBS) were inoculated into the right leg on day 0 and into the left leg on day 4 of 8-12 week-old 129/SvEv syngeneic female mice (*N*=5, Taconic Biosciences) to create a “primary tumor” and “secondary tumor”, respectively. The primary tumors were irradiated with 3 fractions of 12 Gy each with a PXi X-Rad SmART on days 8, 9, and 10. This specific dosage regimen has been optimized by our group based on its efficacy in inducing a potent antitumor immune response, as well as its ability to produce an abscopal effect [24, 25]. The dose was delivered with two opposing beams from the AP and PA positions using a 15 mm circular collimator. The dosimetry and treatment planning were performed using the Advanced Treatment Planning software that is supplied by the manufacturer of the irradiator (Precision XRay Corporation, North Branford, CT). All collimators were commissioned by Precision XRay Corporation at the time of installation. Routine output checks are performed using an ion chamber to ensure that the outputs have not changed and that the treatment plans continue to be accurate. For the MerTK ASO administered intraperitoneally, 50 mg/kg of MerTK ASO (MerTK) in 250 μL PBS was injected to the mice on days 1, 2, 3, 4, 7, 8, 9, 10, 14, 18, 21, 25, 28, 32, 35 and 39 *via* intraperitoneal (IP) injection. Either 10 mg/kg of αPD1 was injected on days 5, 8, 11, 14, 21, 28, and 35 or 2.5 mg/kg of the αCTLA4 was injected on days 5, 8, 11, and 14 *via* IP injection. Starting from day 7, the tumors were measured, and the tumor volumes were calculated as V = 0.5 × width^2^ × length. The mice were euthanized when either the primary or the secondary tumors reached 14 mm in width across any dimension. All animal procedures followed the guidelines of the Institutional Animal Care and Use Committee at The University of Texas MD Anderson Cancer Center.

### Tumor processing

Primary and the secondary tumors were harvested on day 16 for NanoString analysis and on day 21 for flow cytometry. The minced tumor tissues were incubated with 250 µg/mL of Liberase and 20 µg/mL DNAse at 37 °C for 30 min. The cells were either labeled by antibodies for flow cytometry analysis or frozen in TRIzol for RNA extraction.

### Multiplex immune fluorescence staining

The secondary tumors harvested on day 21 were also fixed with 10% formalin and processed and embedded in paraffin routinely. 4 µm sections were cut with a Leica microtome and tissue was stained with H&E. TMA slides created consisting of 3 mm cores from each tissue, followed by staining for CD8 T cell, CD4 T cell, FOXP3, GranzymB, and NKP46, PANCK, and DAPI with an Akoya Bioscience Opal Polaris 7-color kit utilized on a Leica BondRx autostainer. Slides were scanned using a Leica Versa 8 digital fluorescent slide scanner. Biomarkers were quantified using a cellular immunofluorescence algorithm in Leica digital image analysis software.

### Analysis of immune cell populations through flow cytometry

The cells from the tumors harvested on day 21 were labeled with αCD45-PerCP Cy5.5, αCD4-PE/Dazzle594, αCD8-FITC, αGrB-Pacific Blue, αGr1-BV510, αCD11b-APC Fire750, αF4/80-Alexa Fluor 700, αCD38-PE-Cy7, αCD206-PE, αMertK-APC. Samples were measured with a Gallios Flow Cytometer (Beckman Coulter) and analyzed with Kaluza software Version 2.1.

### Analysis of immune-related gene expression *via* nanostring

Total RNA was extracted from the tumors harvested on day 16 with the chloroform/phenol method [26]. The isolated RNA was analyzed with the nCounter PanCancer Immune Profiling Panel and nCounter MAX Analysis System (both from NanoString Technologies, Seattle, WA, USA) according to the manufacturer’s instructions. The data were analyzed with the PanCancer Immune Profiling Advanced Analysis Module.

### Pathological examination of eyes

Eyes from the mice in groups of Control, XRT+MerTK, XRT+MerTK+αPD1, XRT+MerTK+αCTLA4 were harvested on day 21. These tissues were fixed in 10% neutral buffered formalin and then transferred to 70% alcohol after 24-48 hours of fixation. Tissue was routinely processed and embedded in paraffin. Sections were cut at 4 µm and placed on glass slides. H&E and toluidine blue stained slides were submitted for evaluation for toxic changes to the eye, with specific focus on the retinal pigment epithelium. Slides were evaluated by a board-certified veterinary pathologist using a Leica DM2500 microscope. Images of the retinal pigment epithelium were captured at 63x magnification using a Leica DFC495 digital camera and LAS imaging software.

### Statistical analyses

All data were statistically analyzed with Prism 8.0 (GraphPad Software). Tumor growth curves were compared by two-way ANOVA and were presented as mean tumor volume ± standard error of the mean (SEM). Mouse survival rates were analyzed with the Kaplan–Meier method, and estimates were compared with log-rank tests. All the other data were compared with two-tailed t-tests and expressed as mean value ± SEM. *P* values of <0.05 were considered statistically significant.

## Results

### MerTK ASO improves antitumor efficacy of anti-PD1-based immunoradiotherapy

**Fig. 1:**
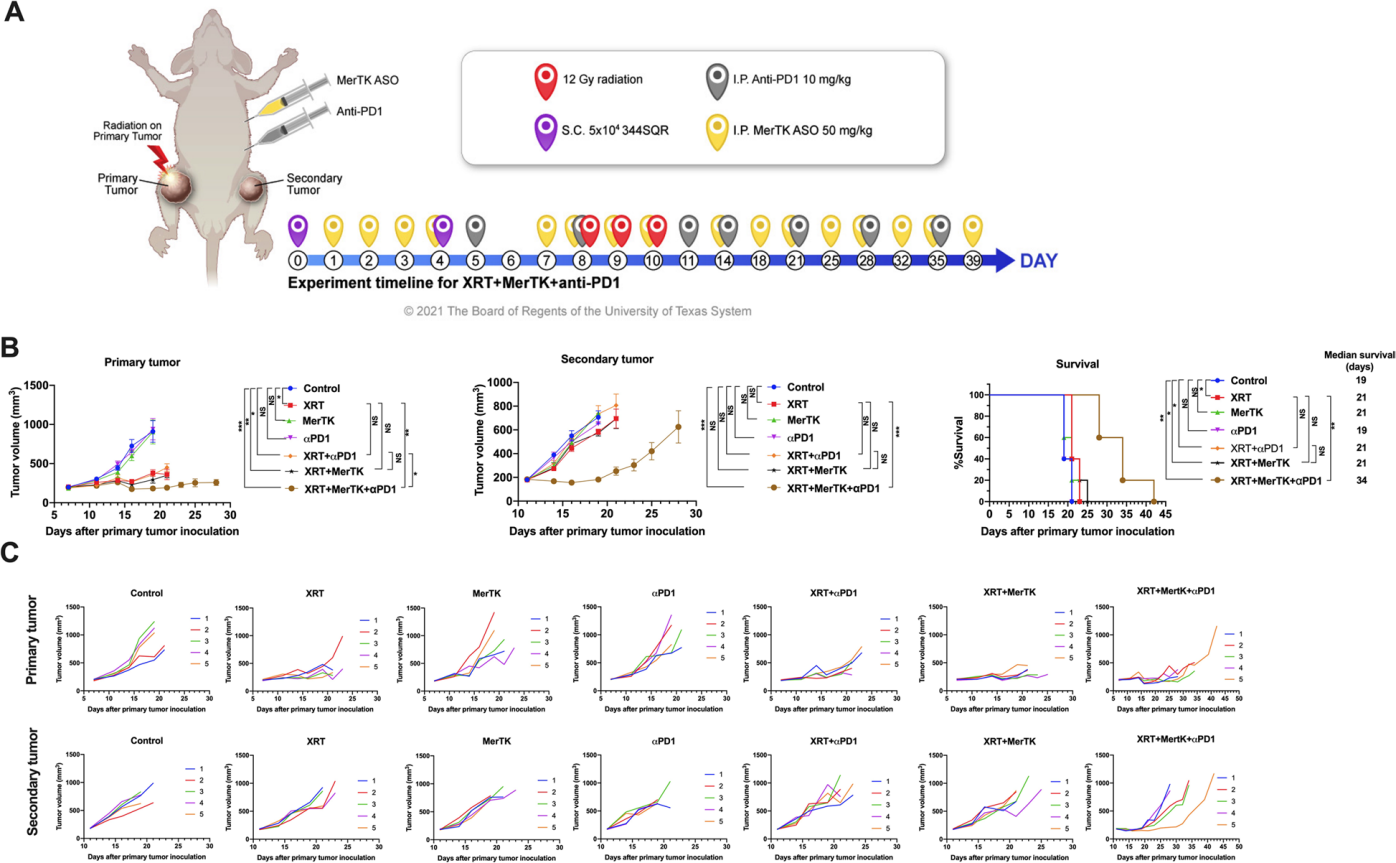
Combination of XRT, MerTK ASO, and αPD1 delays the growth of both the primary and the secondary tumors. **A** Treatment scheme for mice with 344SQR two tumor models. **B** Average tumor volumes and survival curves. **C** Individual tumor growth curves. The mice were inoculated with primary tumors and secondary tumors on the right and left legs on days 0 and 4, respectively. The primary tumors were irradiated with 3x12 Gy radiation on days 8, 9, and 10. The mice were intraperitoneally administered with 10 mg/kg αPD1 and 50 mg/kg MerTK ASO on the indicated time points in Fig. 1A. The tumor volumes were monitored from day 7 and the mice were sacrificed when any dimension of the tumors reached 14 mm. The tumors volumes were compared with two-way ANOVA and expressed as Mean±SEM. The survival curves were compared with log-rank tests. *P* values of <0.05 indicates statistical significance. *P<0.05, **P<0.01, ***P<0.001, ****P<0.0001, NS denotes not significant

We previously showed that blocking MerTK with an antibody can enhance the effects of immunoradiotherapy, but at the cost of retinal toxicity [4, 18]. To avoid deleterious effects to the eyes, we evaluated an antisense oligonucleotide (ASO) that specifically knocks down MerTK expression on tumor growth in combination with localized radiation (XRT) and αPD1 in a mouse model of anti-PD1-resistant cancer (Fig. 1A). The addition of the MerTK ASO to the combination of XRT+αPD1 significantly improved treatment efficacy in both the primary and secondary tumors (Fig. 1B, C). Triple therapy of XRT+αPD1+MerTK ASO (hereafter RPM) achieved a median survival of 34 days, significantly longer than the 21 days achieved with the XRT+αPD1 group. Consistent with our previously reported data, XRT+αPD1 alone was unable to achieve an abscopal effect in the 344SQR model [25]. This was also the case for XRT, αPD1, or MerTK ASO alone, or for XRT+MerTK (Fig. 1B, C, Supplemental Fig. S1). However, RPM was able to produce an abscopal effect. This effect was not achieved when the MerTK ASO was substituted for a control non-targeting ASO (Supplemental Fig. S1).

### MerTK ASO improves efficacy of anti-CTLA4-based immunoradiotherapy

**Fig. 2:**
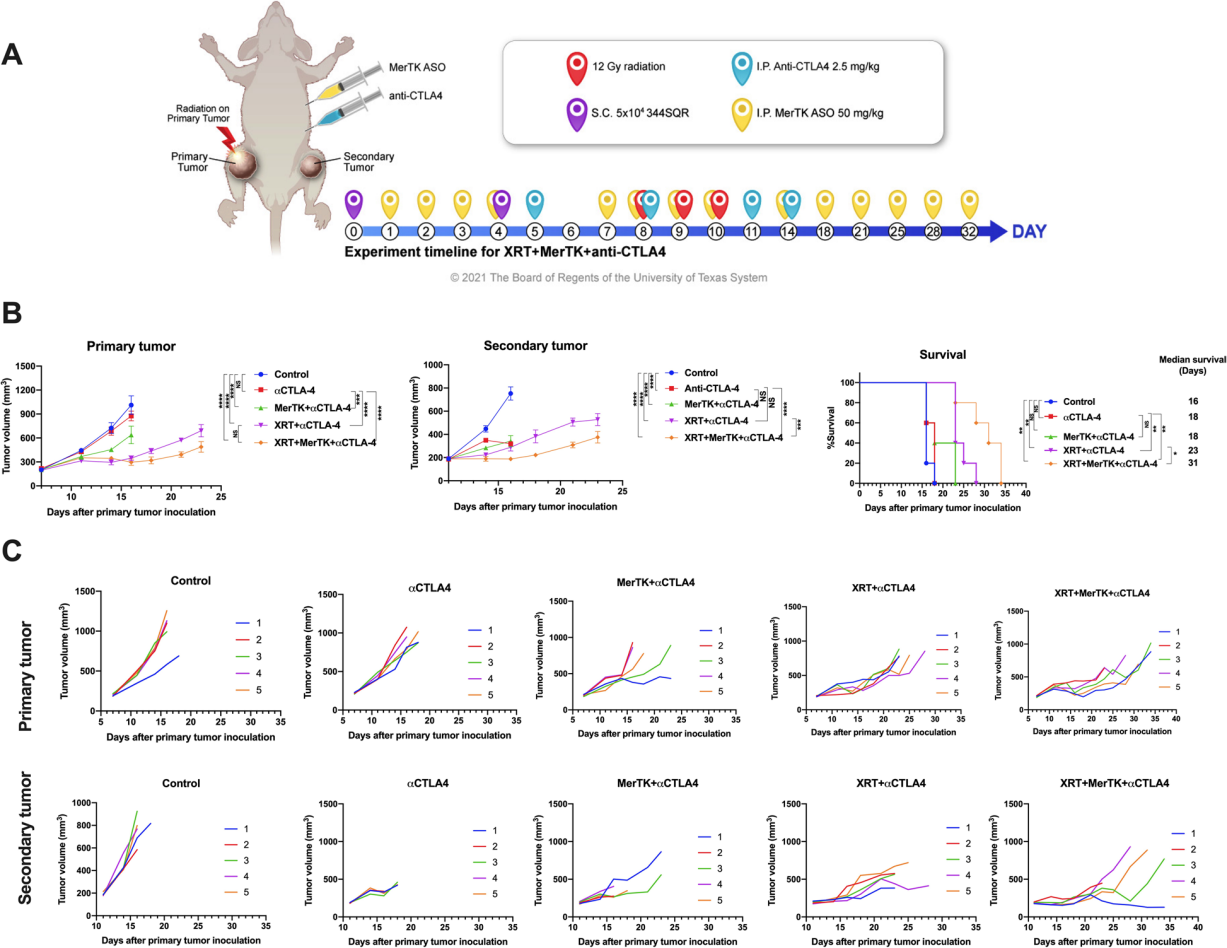
Triple combination of XRT, MerTK ASO, and anti-CTLA4 achieved a better abscopal effect than the dual combination of XRT, MerTK ASO, and anti-CTLA4. **A** Treatment scheme for mice with 344SQR two tumor models. **B** Average tumor volumes and survival curves. **C** Individual tumor growth curves. The mice were inoculated with primary tumors and secondary tumors on the right and left legs on days 0 and 4, respectively. The primary tumors were irradiated with 3x12Gy radiation on day 8, 9, and 10. The mice were intraperitoneally administered with 2.5 mg/kg anti-CTLA4 and 50 mg/kg MerTK ASO on the indicated time points in Fig. 2A. The tumor volumes were monitored from day 7 and the mice were sacrificed when any dimension of the tumors reached 14 mm. The tumors volumes were compared with two-way ANOVA and expressed as mean±SEM. The survival curves were compared with log-rank tests. *P* values of <0.05 indicates statistical significance. *P<0.05, **P<0.01, ***P<0.001, ****P<0.0001, NS denotes not significant

Given the observed efficacy of the MerTK ASO when paired with XRT+αPD1, we sought to determine whether comparable improvements in treatment efficacy could be achieved by the addition of MerTK and XRT to the other first-line checkpoint inhibitor (CPI), αCTLA4 [27, 28]. As shown in Fig. 2A, mice bearing 344SQR tumors were treated with XRT, αCTLA4, and MerTK ASO at the indicated time points. In agreement with previous clinical and mouse studies [29–32], the dual therapy of XRT+αCTLA4 alone was able to achieve an abscopal effect in our mouse tumor model (Fig. 2B and C). The addition of the MerTK ASO to XRT+αCTLA4 (hereafter RCM) significantly enhanced the abscopal effect and extended the median survival to from 23 to 31 days. However, the effects of MerTK ASO did not markedly improve treatment outcome in the primary tumors. Interestingly, dual therapy of MerTK+αCTLA4 without XRT also significantly delayed the growth of both the primary and secondary tumors, albeit not to the same extent achieved with the addition of radiation therapy (Fig. 2B and C). These results indicate the improvement in immune response achieved when pairing MerTK inhibition with immunoradiotherapy is not specific to PD1 blockade, but can be generalized to multiple different CPIs. They also demonstrate that superior tumor control is consistently achieved through the triple combination of MerTK inhibition, XRT, and CPI. In addition to immunoradiotherapy, we also evaluate the therapeutic effect of MerTK ASO when combined with chemotherapy, selumetinib and anti-PDL1, or anti-PD1 in various tumor models. Beyond immunoradiotherapy, we assessed the therapeutic efficacy of MerTK ASO in combination with chemotherapy, selumetinib, and either anti-PDL1 or anti-PD1 across different tumor models (Supplemental Fig. S2). The addition of MerTK ASO to the carboplatin+paclitaxel regimen markedly enhanced tumor control in the 344SQR model (Supplemental Fig. S2A). Incorporating MerTK ASO with AZD6244 (selumetinib) and anti-PDL1 significantly enhanced tumor control (Supplemental Fig. S2B). Furthermore, in the EO771 breast cancer model, the combination of MerTK ASO and anti-PD1 resulted in significantly better tumor control compared to monotherapies (Supplemental Fig. S2C).

### MerTK ASO inhibits MerTK expression on TAMs and promotes M2-to-M1 polarization

**Fig. 3:**
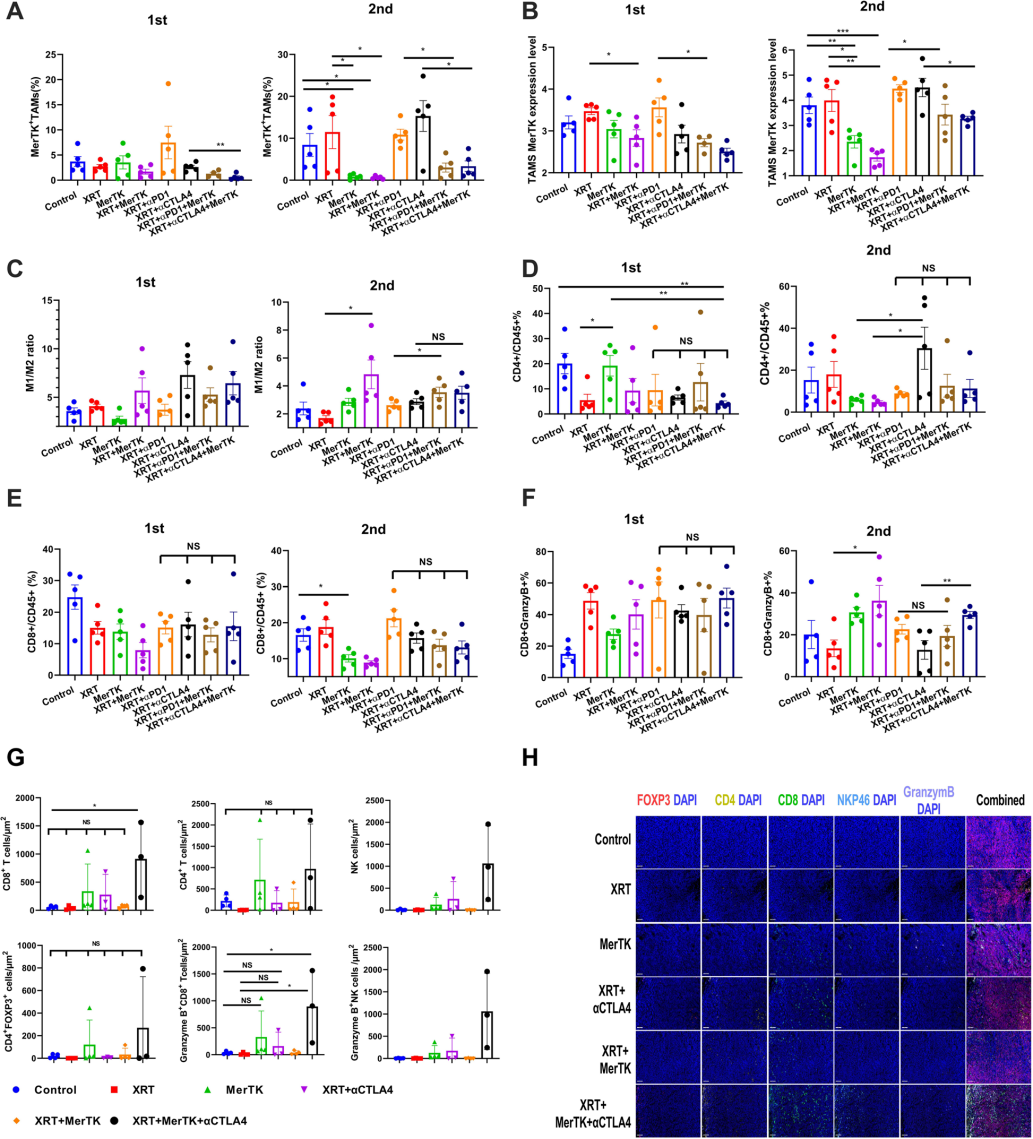
MerTK ASO reshapes the population of immune cells in TME in favor of antitumor immune response **A** The percentages of MerTK^+^ tumor-associated macrophages (TAMs). **B** The MerTK expression level in TAMS. **C** M1/M2 ratio. **D** CD4^+^ T cell/CD45^+^ cell ratio. **E** CD8^+^ T cell/CD45^+^ cell ratio. **F** The percentages of GrB^+^ CD8^+^ T cells out of total CD8^+^ T cells. **G** Densities of CD8^+^, CD4^+^, NK, CD4^+^FOXP3^+^, GranzymB^+^CD8^+^, and GranzymB^+^NK cells in the secondary tumors stained with multiplex immune flouorescence. **H** Representative multiplex immune fluorescence images of Fig. G. The mice were treated with different combinations of XRT, MerTK ASO, αPD1, and αCTLA4, as indicated in Fig. 1A and Fig. 2A, and were sacrificed on day 21. Both the primary and the secondary tumors were harvested and stained with αCD45-PerCP-Cy5.5, αCD4-PE/Dazzle594, αCD8-FITC, αGrB-Pacific Blue, αGr1-BV510, αCD11b-APC Fire750, αF4/80-Alexa Fluor 700, αCD38-PE-Cy7, αCD206-PE, αMertK-APC. The secondary tumors tissues from groups Control, XRT, MerTK, XRT+αCTLA4, XRT+MerTK, XRT+MerTK+ αCTLA4 were also fixed and processed for multiplex immune fluoresce and further stained with DAPI, FOXP3, CD4, CD8, NKP46, and granzymB. All the statistics were compared with two-tailed t tests and expressed as mean value ± SEM. *P* values of <0.05 indicates statistical significance. *P<0.05, **P<0.01, ***P<0.001, NS denotes not significant

Mice were conceptually divided into one of four treatment groups: 1) Control, 2) XRT, 3) XRT+ αPD1and 4) XRT+αCTLA4. In each of these four groups was divided into two subgroups – one which received the MerTK ASO, and one which did not. We harvested the tumors of these mice on day 21, and the immune cells therein were analyzed *via* flow cytometry to evaluate the effect the MerTK ASO had on each treatment (Fig. 3, Supplemental Fig. 3). The addition of the MerTK ASO to XRT+ αCTLA4 significantly decreased the percentage of MerTK positive TAMs in the primary tumors (Fig. 3A). The MerTK ASO had no effect on MerTK expression level on TAMs in the primary tumors of control mice (Fig. 3B). However, the addition of the MerTK ASO to either XRT or XRT+αPD1 significantly reduced MerTK expression level on TAMs in primary tumors (Fig. 3B). In the secondary tumors, this effect was even more stark, with the MerTK ASO significantly reducing MerTK expression in all four treatment groups (Fig. 3A and B, Supplemental Fig. 3). This dramatic reduction in MerTK expression on the TAMs in the secondary tumor was accompanied by an increase in the M1/M2 ratio, which reached statistical significance for the XRT and XRT+αPD1 treatment groups (Fig. 3C). We next looked at the presence of CD45^+^ tumor-infiltrating lymphocytes. The prevalence of CD4^+^CD45^+^ T cells was unaffected by MerTK inhibition in either tumor (Fig. 3D), as was the prevalence of CD8^+^CD45^+^ T cells in the primary tumor (Fig. 3E). CD8^+^CD45^+^ T cell prevalence actually decreased following MerTK inhibition in the secondary tumors. However, the percentage of granzyme B^+^ (granzB^+^) CD8^+^ T cells significantly increased in the secondary tumors of mice treated with XRT+MerTK ASO or XRT+αCTLA4+MerTK ASO (Fig. 3F). In the primary tumors, MerTK alone produced significant elevation in the percentage of granzB^+^CD8^+^ T cells compared to the control mice, but there was no significant change between any of the other treatment groups within the primary tumor (Fig. 3F). Of note was that there was no change in the percentage of granzB^+^CD8^+^ T cells between the XRT+αPD1 and RPM groups in either the primary or the secondary tumor. In addition, the densities of CD8 T cells, CD4 T cells, NK cells, and Tregs as well as the granzymB+CD8+ T cells and granzymB+NK cells in the secondary tumors were quantified by multiplex immune fluorescences staining (Fig. 3G and H). Significantly more CD8 T cells and granzymB^+^ CD8 T cells were observed only in the XRT+ XRT+αCTLA4+MerTK group in relative to the control group. No significant difference were detected in densities of CD4 T cell, NK, cell, Tregs, and granymB^+^NK cells among all the treatment groups.

### MerTK ASO modulates the expression of immune-related genes in the primary tumor

**Fig. 4:**
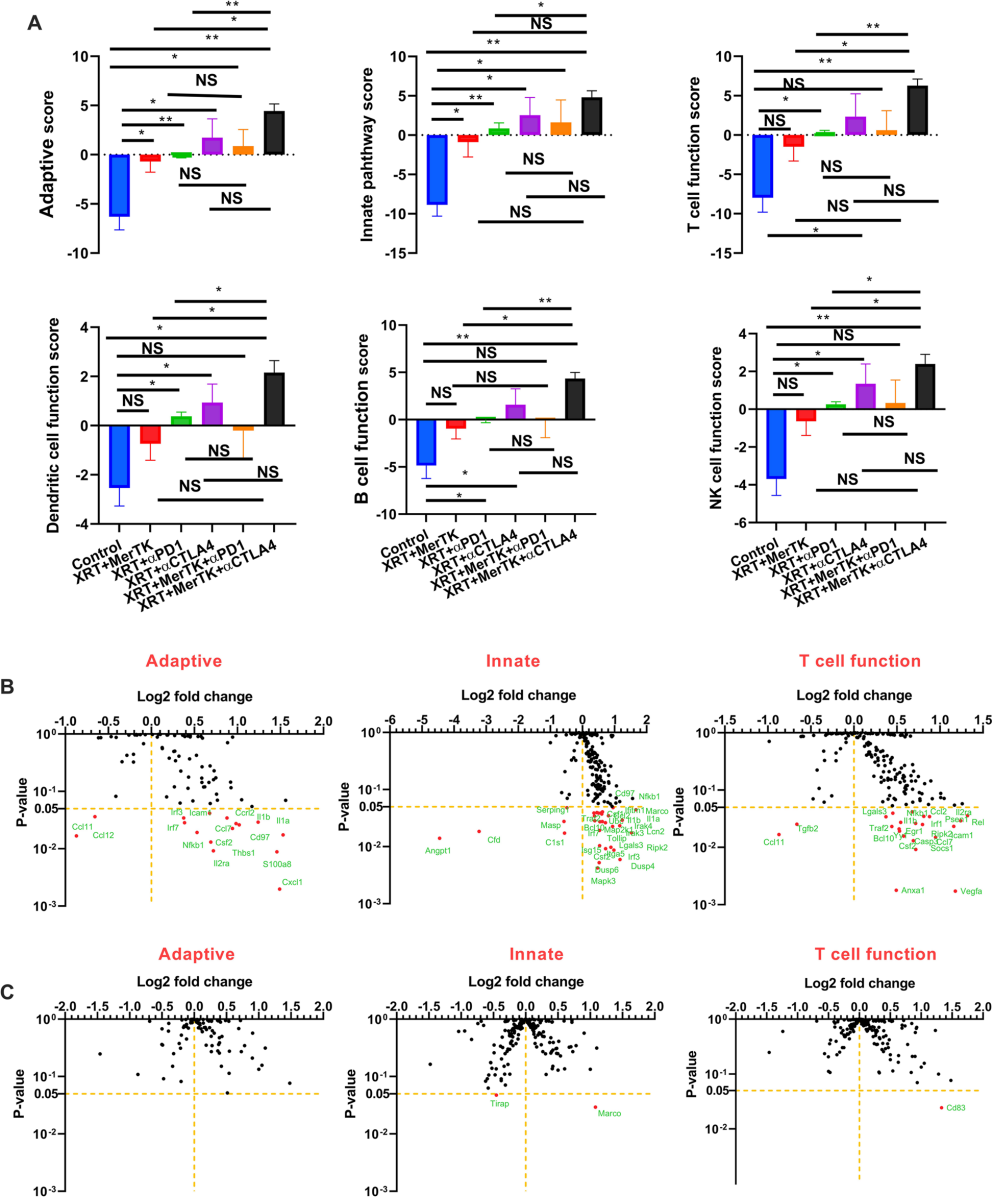
MerTK ASO modulates the expression of immune-related genes in the primary tumors. **A** Scores of various immune pathways in the primary tumors of the mice treated with XRT+MerTK, XRT+αPD1, XRT+αCTLA4, RPM, and RCM. **B** Changes in expression of genes in adaptive pathway, innate pathway, and T cell function of XRT+αPD1+MerTK in relative to XRT+αPD1. **C** Changes in expression of genes in adaptive pathway, innate pathway, and T cell function of XRT+αCTLA4+MerTK in relative to XRT+αCTLA4. The mice (*n*=3) were treated with different combinations of XRT, MerTK ASO, αPD1, and αCTLA4, as indicated in Fig. 1A and Fig. 2A, and were sacrificed on day 16. The total RNA extracted from the primary tumors was analyzed with a nCounter PanCancer Immune Profiling Panel. All the statistics were compared with two-tailed t tests and expressed as mean value ± SEM. *P* values of <0.05 indicates statistical significance. *P<0.05, **P<0.01, NS denotes significant

To gain broader insight as to what changes were occurring at the mRNA level in response to MerTK inhibition, we examined the impact of the ASO’s administration on the expression of immune-related gene transcripts isolated from the primary tumors *via* NanoString. The isolated RNA was analyzed with the nCounter PanCancer Immune Profiling Panel and Advanced Analysis Module, which assigns the identified transcripts to particular pathways, which are, in turn, scored based upon the abundance of the constituent transcripts. Using this analysis, we observed that all treatment groups demonstrated significantly higher activities compared to the control group in both the adaptive and innate immune pathways (Fig. 4A). The combination of XRT, MerTK ASO and αCTLA4 produced significantly higher activities in all the immune pathways compared to the control (Fig. 4A, Supplemental Fig. S4A). In addition, the immune cell scores in Supplemental Fig. S5 demonstrate that the mice treated with combinations of XRT, MerTK ASO, αPD1, and αCTLA4 had a seemingly higher abundance of neutrophils, CD8^+^ T cells, dendritic cells (DCs), macrophages, and CD45^+^ cells in the primary tumor. However, within the primary tumor, other than a slight increase in T_H_1 cells in mice treated with RCM (Supplemental Fig. S5), there was no additional activation of any immune pathways gained from adding the MerTK ASO to any other therapeutic modality (Fig. 4A, Supplemental Fig. S4A).

When comparing XRT+aCTLA4 to RCM, there was virtually no change in the expression of any individual genes (Fig. 4B, Supplemental Fig. S4B ). In contrast, there was a slight shift in a number of genes between XRT+aPD1 and RPM, all <2 log_2_ fold. Examining the most upregulated genes showed evidence of innate immune activation and mobilization, with upregulation of genes such as: *Marco*, a macrophage-tropic pattern-recognition receptor that recognizes low-density lipoproteins; *Il1a,* a cytokine produced by monocytes and macrophages that stimulates B and T cell proliferation; *Cxcl1,* a chemoattractant for neutrophils; *S100a8,* one of the two components of calprotectin secreted by monocytes, granulocytes, and neutrophils during inflammation; *Rel,* the gene for c-Rel, an NFκB family member important for B cell development; and *Il1b*, one of the two primary inflammatory cytokines produced by activation of the inflammasome. Altogether, this suggests that the immune response stimulated by MerTK inhibition at the primary tumor following immunoradiotherapy is primarily innate in nature.

### MerTK ASO promotes a broad antitumor immune response in the secondary tumors

**Fig. 5:**
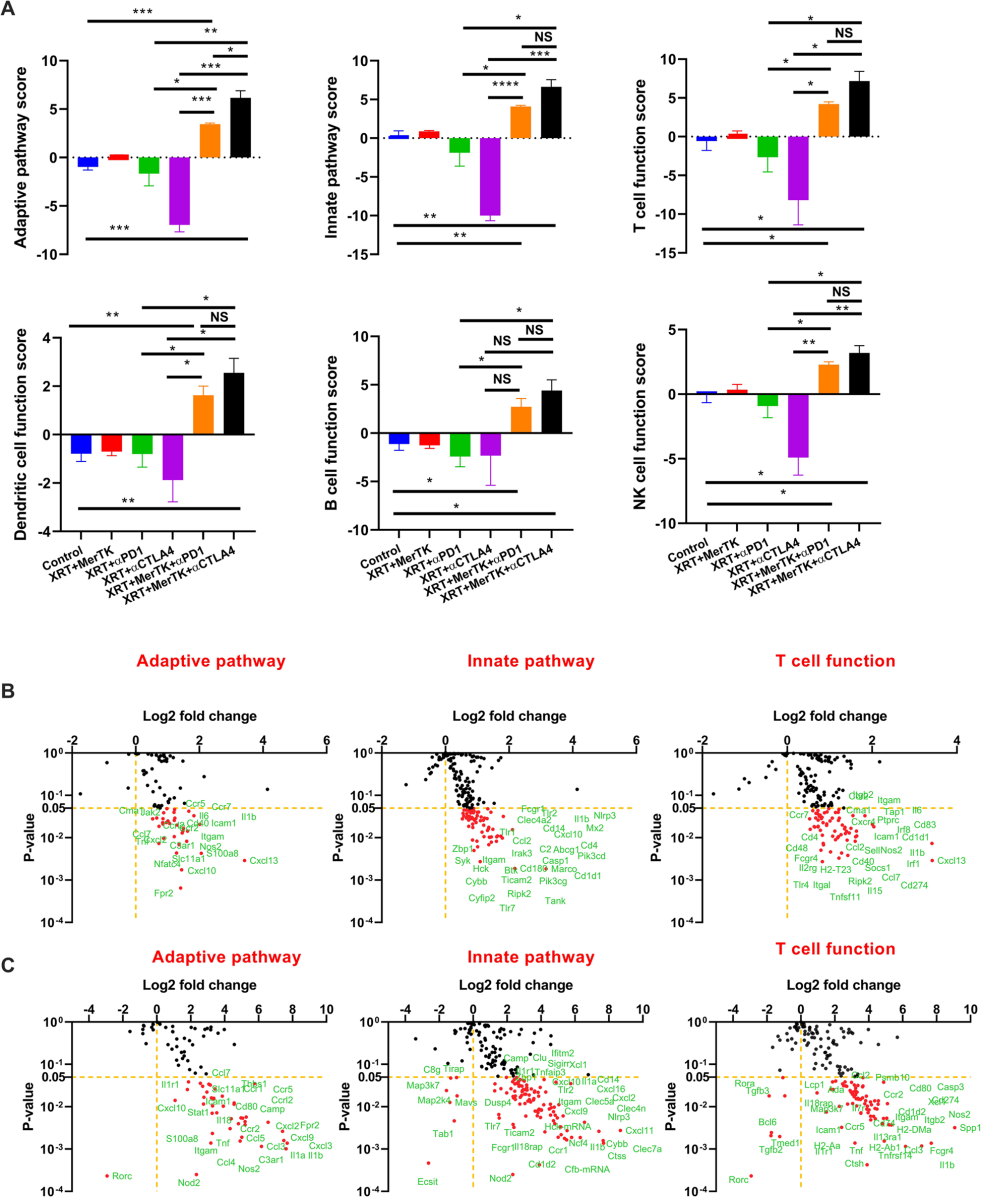
MerTK ASO modulates the expression of immune-related genes in the secondary tumors. **A** Scores of various immune pathways in the secondary tumors of the mice treated with XRT+MerTK, XRT+αPD1, XRT+αCTLA4, RPM, and RCM. **B** Changes in expression of genes in adaptive pathway, innate pathway, and T cell function of XRT+αPD1+MerTK in relative to XRT+αPD1. **C** Changes in expression of genes in adaptive pathway, innate pathway, and T cell function of XRT+αCTLA4+MerTK in relative to XRT+αCTLA4. The mice (*n*=3) were treated with different combinations of XRT, MerTK ASO, αPD1, and αCTLA4, as indicated in Fig. 1A and Fig. 2A, and were sacrificed on day 16. The total RNA extracted from the secondary tumors was analyzed with a nCounter PanCancer Immune Profiling Panel. All the statistics were compared with two-tailed t tests and expressed as mean value ± SEM. *P* values of <0.05 indicates statistical significance. *P<0.05, **P<0.01, NS denotes significant. All statistics were done using two-tailed t tests and expressed as mean value ± SEM. *P* values of <0.05 indicates statistical significance. *P<0.05, **P<0.01, ***P<0.001, NS denotes not significant

Given the lack of any substantive increase in immune pathway activation in the primary tumor induced by adding the MerTK ASO to immunoradiotherapy, we examined these same pathways in the secondary tumor. As shown in Fig. 5A and Supplemental Fig. S6A, the addition of the MerTK ASO to XRT+αPD1 and XRT+αCTLA4 significantly promoted the activities of all measured immune pathways, including adaptive and innate immunity, antigen processing, T cell function, *etc*. In addition, neither XRT+αPD1, nor XRT+αCTLA4, nor XRT+MerTK induced higher immune activities compared to the control in the secondary tumors. However, the combination of RPM or RCM demonstrated significantly increased activities in all the immune pathways compared with the control.

Furthermore, changes in the expression of individual genes caused by the addition of MerTK ASO to XRT+αPD1 and XRT+αCTLA4 were analyzed. As shown in Fig. 5B and Supplemental Fig. S6B, genes involved in B cell, T cell, macrophage, and NK cell function were all upregulated in the secondary tumors of mice treated with the MerTK ASO. We then further analyzed those genes found by the nCounter analysis to be significantly upregulated in the RPM and RCM groups relative to the XRT+CPI groups. These genes were manually grouped into pathways in which they were known to be involved. Doing this, we found that genes in several specific immune-related pathways were upregulated within the secondary tumors of mice treated with the MerTK ASO, including ROS generation, pattern recognition receptors signaling, complement and humoral immunity, adhesion and cell-cell interactions, inflammation, and NFκB signaling. This relative immune gene upregulation was, by far, the most dramatic in the secondary tumors of mice treated with RCM (Supplemental Fig. S7); compared to mice treated with XRT+αCTLA4 alone, mice treated with RCM exhibited a two-fold increase in the expression of several immune-related genes across multiple different pathways. The immune activation gained from the addition of the MerTK ASO to mice treated with XRT+αPD1 was more modest but still substantial.

In both cases (αPD1 and αCTLA4), the immune activation at the secondary tumor was much greater than that at the primary tumor. These results may suggest that control of the primary tumor is principally mediated through the direct effects of the radiotherapy, whereas control of the secondary tumor in our model system is principally mediated by the immune system, which has been invigorated by the conversion of the radiation-damaged primary tumor into an *in situ* vaccine [33], and then further bolstered through the activity of MerTK inhibition.

In addition, as shown in Supplemental Fig. S8, the addition of MerTK ASO to XRT+αPD1 or XRT+αCTLA4 considerably increased the abundance of neutrophils, CD8^+^ T cells, DCs, NK cells, macrophages, and CD45^+^ cells in the secondary tumors. Again, RCM seemed to result in more T_H_1 cells than XRT+αCTLA4. Given the higher M1/M2 ratio seen in mice treated with RPM and RCM shown in Fig. 3C, we believe that the increased numbers of macrophages within the tumors of mice treated with MerTK ASO-augmented immunoradiotherapy is more likely to promote immune activation rather than suppression.

### Integration of MerTK ASO to immunoradiotherapy does not cause retinal toxicity

**Fig. 6:**
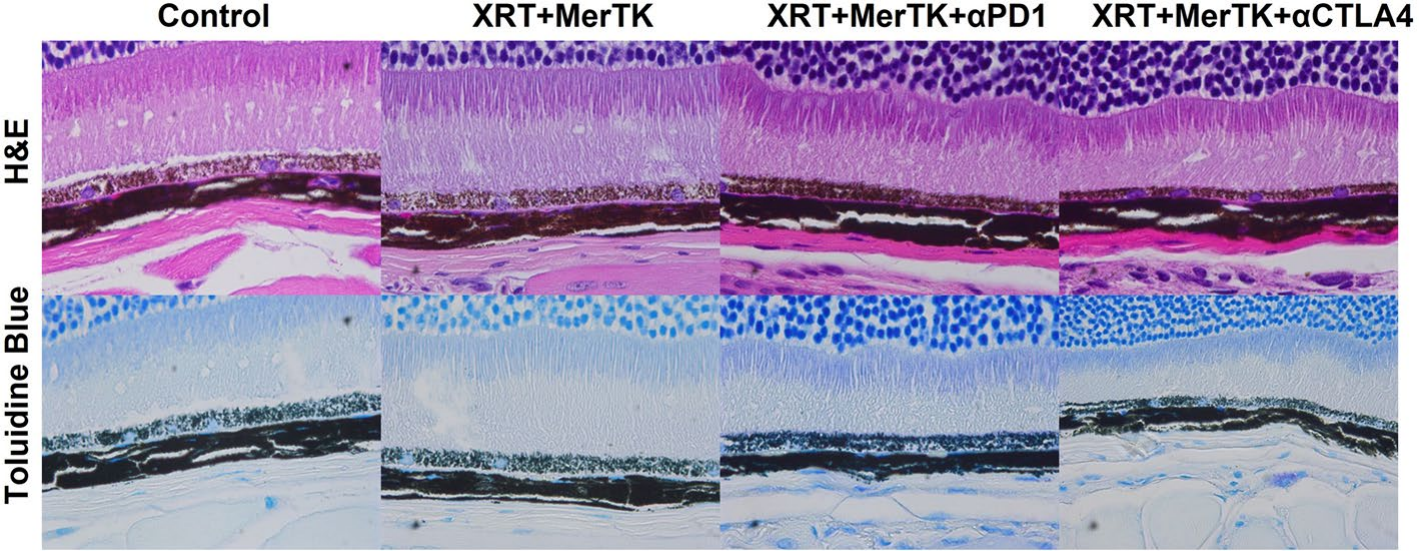
MerTK ASO causes no retinal toxicity. Eyes from mice were harvested on day 21 and stained with H&E and toluidine blue

Prior studies have established that MerTK inhibition via small molecules and antibodies compromises the phagocytic functionality of the retina and precipitates morphological alterations in the retinal pigment epithelium [17, 18]. To assess the ocular safety of MerTK ASO, we conducted histological examinations using H&E and toluidine blue staining of eye tissues from both untreated (control) mice and those treated with XRT+MerTK, RPM, RCM. Fig. 6 illustrates that no histological abnormalities were detected in the eye tissues of the treated groups compared to the control group. Furthermore, we evaluated the effects of increasing doses of MerTK ASO on MerTK expression and potential pathological impacts in the eye. Independent of dosage, MerTK ASO did not significantly modify MerTK expression in the murine eye (Supplemental Fig. S9A), nor did it induce noticeable pathological variations compared to the control (Supplemental Fig. S9B).

## Discussion

While radiotherapy-induced apoptosis of cancer cells has been reported to elicit effective systemic antitumor immune responses, MerTK expressed on macrophages may considerably suppress the activation of CD8^+^ T cells by promoting the efferocytosis of apoptotic cells by macrophages [34, 35]. Antibodies and small molecules targeting MerTK have demonstrated great potential in alleviating MerTK-mediated immune suppression, However, these therapies cause severe damage to the retina in preclinical studies [17, 18]. In contrast, our results demonstrate that MerTK ASOs, when combined with localized radiotherapy and CPIs, can promote antitumor activity in mice without retinal toxicity. The addition of the MerTK ASO to the combination of radiotherapy and CPI in our study reliably induced an abscopal effect, with substantial reductions in secondary tumor growth and coordinated improvements in animal survival. This abscopal, anti-tumor effect was lacking in mice treated with XRT+αPD1, suggesting that the MerTK ASO was able to convert αPD1-resistant tumors to αPD1-sensitive tumors.

In this study, we observed a shift in TAM polarization from the pro-tumor M2 phenotype to the anti-tumor M1 phenotype in the secondary tumors of mice treated with XRT+CPI+MerTK ASO. Concomitantly, we observed significantly more tumor infiltrating lymphocytes in both the primary, irradiated and secondary, unirradiated tumors. This, in combination with the upregulation of a wide range of immune pathways in the secondary tumors, illustrates the classical two-step activation of the immune system. Operating within the conceptual framework of radiation turning the tumor into an *in situ* vaccine [33], CPI and MerTK inhibition can be thought of as immune stimulators, each providing independent, synergistic stimulation to the immune system that multiplies its activation following this radiological “vaccination”.

High levels of MerTK TAM expression in secondary tumors were observed in the combination of XRT+αPD1 or XRT+αCTLA4, indicating that these therapeutic modalities may induce high degree of efferocytosis. This aligns with our overall hypothesis that XRT+CPI induces large amounts of tumor cell death and subsequent immune activation, but that the latter is stymied through maladaptive efferocytosis that curtails the inflammatory immune response. When XRT+αPD1 or αCTLA4 was supplemented with the MerTK ASO, however, we observed a dramatic reversal in the MerTK TAM expression level from the highs we observed following treatment with XRT + either CPI. Both of these parameters were reduced to the levels of untreated controls or even lower in both the primary and secondary tumors, and this was the case whether we blocked PD1 or CTLA4. Furthermore, our findings indicate that MerTK ASO can efficiently target TAMs without the necessity for specialized tumor delivery systems like nanoparticles. This attribute potentially facilitates its more straightforward clinical application.

A noteworthy finding of this study is that these resultant immune responses differed in a number of respects depending on whether CPI was done with αPD1 or αCTLA4. This is, of course, to be expected; it is well known that PD1 and CTLA4 mediate their immunosuppressive effects on the immune system through entirely distinct mechanisms [36]. How blockade of each of these combines with radiotherapy and MerTK inhibition, though, is completely novel territory. As previously mentioned, both therapies induced an abscopal effect, showing a strong reduction in both primary and secondary tumor growth. However, the mechanisms by which this effect was achieved differed notably. In mice treated with RPM, there was prominent M2-to-M1 polarization in the secondary tumor; this was not present to a significant degree in mice treated with RCM. Adding the MerTK ASO to XRT+αPD1 made a much bigger difference to tumor control and animal survival than was the case for XRT+αCTLA4, though both triple combinations achieved similar efficacy in the end. In contrast, RCM seemed to promote more T_H_1 CD4^+^ and GrB^+^ CD8^+^ T cell recruitment to the secondary tumors, at least in comparison to XRT+αCTLA4 without the MerTK ASO. Indeed, the difference in overall immune activation between dual therapy and tri-therapy (i.e. XRT+CPI vs. XRT+CPI+MerTK) was much greater for mice treated with αCTLA4 than those treated with αPD1. Overall, both tri-therapies achieved comparable efficacy in our mouse model, suggesting that both of the front-line CPIs in clinical use today can benefit from supplementation with MerTK inhibition.

The integration of MerTK ASO with XRT combined with either αPD1 or αCTLA4 yields distinct therapeutic outcomes, an observation that underscores the nuanced interplay between these treatments and their underlying mechanisms of action. αCTLA4 therapy, by virtue of its action mechanism, initiates a broad activation of T cells during the early phases of the immune response. Conversely, αPD-1 therapy is more targeted in its approach, primarily enhancing the effector functions of T cells that have already recognized tumor antigens. Such specificity is particularly efficacious against tumors exhibiting elevated PD-L1 expression, a condition that can be exacerbated by radiotherapy [37, 38]. MerTK inhibition by ASO, in conjunction with these treatments, further influences the TME by modulating macrophage activity, which plays a critical role in both the innate and adaptive immune responses to cancer.

Our histopathological analysis revealed no significant retinal alterations in mice subjected to escalating doses of MerTK ASO, XRT+MerTK ASO, or the combination of XRT+MerTK ASO+CPIs. This finding suggests that, unlike αMerTK antibodies and small molecule inhibitors, MerTK ASO does not precipitate retinal damage. This attribute potentially enhances its viability for clinical translation.

In this study, we have delved into the synergistic potential of MerTK ASO when combined with radiotherapy and immunotherapy. In clinic, the timing of immunotherapy, including agents like αPD1 and αCTLA4, relative to radiotherapy, is tailored according to the specific type of cancer being treated. The central function of MerTK ASO is to block immune suppression carried out by M2 macrophages. Post-radiation, there is a tendency for macrophages to transition towards an M2 phenotype [39]. Based on this understanding, integrating MerTK ASO either concurrently with or shortly before radiotherapy appears to be the most effective strategy.

## Conclusions

In conclusion, the combination of XRT, MerTK ASO, and CPIs promoted M1 macrophage polarization, facilitated the infiltration of effector immune cells, promoted the activation of CD8^+^ T cells in the unirradiated tumors, and upregulated the expression of multiple immune-related genes involved in anto-tumor activity, and all of which resulted in an improved abscopal effect in an αPD1-resistant tumor. These results reinforce MerTK’s candidacy as a druggable target in cancer and establish the MerTK ASO as a safe and effective means by which to do so.

### Supplementary Information

**Additional file 1:** **Supplemental Fig. S1.** Combination of XRT, MerTK ASO, and anti-PD1 delays the growth of both the primary and the secondary tumors. A, Treatment scheme for mice with 344SQR two tumor model. B, Average tumor volumes and survival curves. C, Individual tumor growth curves. The mice were inoculated with primary tumors and secondary tumors on the right and left legs on days 0 and 4, respectively. Primary tumors were irradiated with 3x12 Gy radiation on days 8, 9, and 10. The mice were intraperitoneally administered with 10 mg/kg anti-PD1 and 50 mg/kg MerTK ASO on the indicated time points in supplemental Fig. 1A. The tumor volumes were monitored from day 7 and the mice were sacrificed when any dimension of the tumors reached 14 mm. The tumors volumes were compared with two-way ANOVA and expressed as mean±SEM. The survival curves were compared with log-rank tests. *P* values of <0.05 indicate statistical significance. *P<0.05, **P<0.01, ***P<0.001, NS denotes not significant.

**Additional file 2:** **Supplemental Fig. S2.** Enhanced therapeutic efficacy of MerTK ASO in combination with chemotherapy, MEK inhibitor, and immune checkpoint inhibitors in diverse tumor models. A, Tumor volume response to combination therapy of carboplatin, paclitaxel, and MerTK ASO in the 344SQR tumor model. Eight-twelve-week old female 129Sv/Ev mice (*n*=5 per group) were subcutaneously injected with 5x10^4^ 344SQR cells in the right leg on day 0. Groups included control, carboplatin+paclitaxel, and carboplatin+paclitaxel+MerTK. Treatments administered were carboplatin (100 mg/kg) and paclitaxel (20 mg/kg) on day 7, followed by paclitaxel on days 9 and 11, anti-PD1 (10 mg/kg) on days 6, 8, 11, and 14, and MerTK ASO (50 mg/kg) on days 7-11 and 14-18. B, Tumor volume response to combination therapy of AZD6244, anti-PDL1, and MerTK ASO. Eight-twelve-week old female 129Sv/Ev mice were assigned to control (*n*=5), AZD6244+anti-PDL1 (*n*=5), or AZD6244+MerTK+anti-PDL1 (*n*=8) groups and were subcutaneously injected with 5x10^5^ 393P cells in the right leg on day 0. The regimen included MerTK ASO (50 mg/kg) on days 14-18 and 21-25, AZD6244 (25 mg/kg) on days 14-18, and anti-PDL1 (10 mg/kg) on days 17, 25, and 32. C, Tumor volume response to combinations of MerTK ASO and anti-PD1 in the EO771 breast cancer model. Eight-twelve-week old female C57BL/6 mice (*n*=8 per group) received subcutaneous injections of 1x10^5^ EO771 cells on days 0 and 4 to establish primary and secondary tumors in the right and left legs, respectively. The treatment groups included control, anti-PD1, MerTK, and MerTK+anti-PD1. Treatment protocol involved MerTK ASO (50 mg/kg) on days 7-11 and 14-18, and anti-PD1 (10 mg/kg) on days 14, 17, and 21.

**Additional file 3:** **Supplental Fig. S3.** Representative FACS images of MerTK^+^ macrophages. A, Primary tumors. B, Secondary tumors.

**Additional file 4:** **Supplemental Fig. S4.** MerTK ASO modulates the expression of immune-related genes in the primary tumors. A, Scores of various immune pathways in the primary tumors of the mice treated with XRT+MerTK, XRT+αPD1, XRT+αCTLA4, RPM, and RCM. B, Changes in expression of genes in the macrophage function, B cell function and NK cell function of XRT+αPD1+MerTK in relative to XRT+αPD1. C, Changes in expression of genes in the macrophage function, B cell function and NK cell function of XRT+αCTLA4+MerTK in relative to XRT+αCTLA4. The mice (*n*=3) were treated with different combinations of XRT, MerTK ASO, αPD1, and αCTLA4, as indicated in Fig.1A and Fig. 2A, and were sacrificed on day 16. The total RNA extracted from the primary tumors was analyzed with a nCounter PanCancer Immune Profiling Panel. All the statistics were compared with two-tailed t tests and expressed as mean value ± SEM. *P* values of <0.05 indicates statistical significance. *P<0.05, **P<0.01, NS denotes significant.

**Additional file 5:** **Supplemental Fig. S5.** NanoString scores of various immune cells in the primary tumors. The mice (*n*=3) were treated with different combinations of XRT, MerTK ASO, anti-PD1, and anti-CTLA4 as indicated in Fig. 1A and Fig. 2A and were sacrificed on day 16. The total RNA extracted from the primary tumors was analyzed with a nCounter PanCancer Immune Profiling Panel. All the statistics were expressed as mean value ± SEM.

**Additional file 6:** **Supplemental Fig. S6.** MerTK ASO modulates the expression of immune-related genes in the secondary tumors. A, Scores of various immune pathways in the primary tumors of the mice treated with XRT+MerTK, XRT+αPD1, XRT+αCTLA4, RPM, and RCM. B, Changes in expression of genes in the macrophage function, B cell function and NK cell function of XRT+αPD1+MerTK in relative to XRT+αPD1. C, Changes in expression of genes in the macrophage function, B cell function and NK cell function of XRT+αCTLA4+MerTK in relative to XRT+αCTLA4. The mice (*n*=3) were treated with different combinations of XRT, MerTK ASO, αPD1, and αCTLA4, as indicated in Fig.1A and Fig. 2A, and were sacrificed on day 16. The total RNA extracted from the secondary tumors was analyzed with a nCounter PanCancer Immune Profiling Panel. All the statistics were compared with two-tailed t tests and expressed as mean value ± SEM. *P* values of <0.05 indicates statistical significance. *P<0.05, **P<0.01, NS denotes significant.

**Additional file 7:** **Supplemental Fig. S7.** MerTK ASO significantly changed the activity of various pathways in the secondary tumor when added to XRT+aPD1 and XRT+aCTLA4.

**Additional file 8:** **Supplemental Fig. S8.** Nanostring scores of various immune cells in the secondary tumors. The mice (*n*=3) were treated with different combinations of XRT, MerTK ASO, anti-PD1, and anti-CTLA4 as indicated in Fig. 1A and Fig. 2A and were sacrificed on day 16. The total RNA extracted from the primary tumors was analyzed with an nCounter PanCancer Immune Profiling Panel. All statistics were expressed as mean value ± SEM.

**Additional file 9:** **Supplemental Fig. S9.** Evaluation of dose-dependent effects of MerTK ASO on MerTK expression and ocular pathology. Female 129Sv/Ev mice aged 8-12 weeks (*n*=8 per group) received treatments with varying dosages of MerTK ASO: 50 mg/kg on days 8 and 12 (total 2x50 mg/kg), 50 mg/kg on days 1-5, 8-12, 15, and 19, 100 mg/kg on days 1-5, 8-12, 15, and 19, and 200 mg/kg on days 8 and 12. On day 22, the mice were euthanized, and their eyes were excised. MerTK expression was analyzed via RT-PCR. Additionally, the eyes underwent Hematoxylin & Eosin (H&E) staining to assess potential pathological alterations.

## Data Availability

The data produced in this study can be made available upon request by contacting the corresponding author.

## Abrreviations

ASO: Antisense oligonucleotide
MerTK: MER proto-oncogene tyrosine kinase
TAMs: Tumor-associated macrophages
TGFβ: Transforming growth factor-β
HGF: Hepatocyte growth factor
TME: Tumor microenvironment
NSCLC: Non-small cell lung cancer
XRT: Radiation
PBS: Phosphate-buffered saline
SEM: Standard error of the mean
CPI: Checkpoint inhibitor
