# Supplementary figures and images for: Inhibition of MER proto-oncogene tyrosine kinase by an antisense oligonucleotide enhances treatment efficacy of immunoradiotherapy
Source: J Exp Clin Cancer Res. 2024 Mar 6;43:70. doi: 10.1186/s13046-024-02992-2 (PMC10916163; doi:10.1186/s13046-024-02992-2)

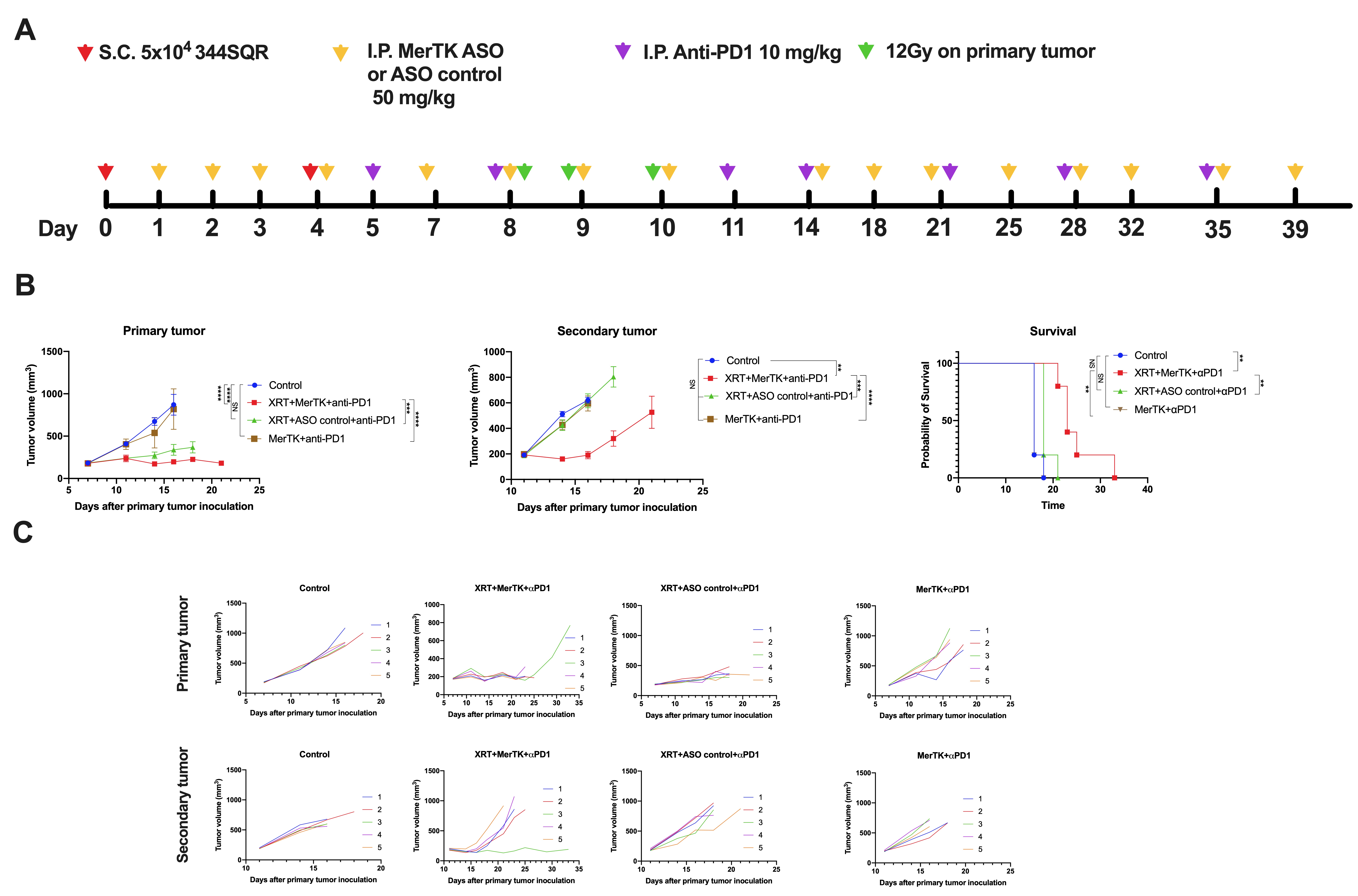

Supplement: Supplementary file 1 — Additional file 1: Supplemental Fig. S1. Combination of XRT, MerTK ASO, and anti-PD1 delays the growth of both the primary and the secondary tumors. A, Treatment scheme for mice with 344SQR two tumor model. B, Average tumor volumes and survival curves. C, Individual tumor growth curves. The mice were inoculated with primary tumors and secondary tumors on the right and left legs on days 0 and 4, respectively. Primary tumors were irradiated with 3x12 Gy radiation on days 8, 9, and 10. The mice were intraperitoneally administered with 10 mg/kg anti-PD1 and 50 mg/kg MerTK ASO on the indicated time points in supplemental Fig. 1A. The tumor volumes were monitored from day 7 and the mice were sacrificed when any dimension of the tumors reached 14 mm. The tumors volumes were compared with two-way ANOVA and expressed as mean±SEM. The survival curves were compared with log-rank tests. P values of <0.05 indicate statistical significance. *P<0.05, **P<0.01, ***P<0.001, NS denotes not significant. [file 13046_2024_2992_MOESM1_ESM.jpg]

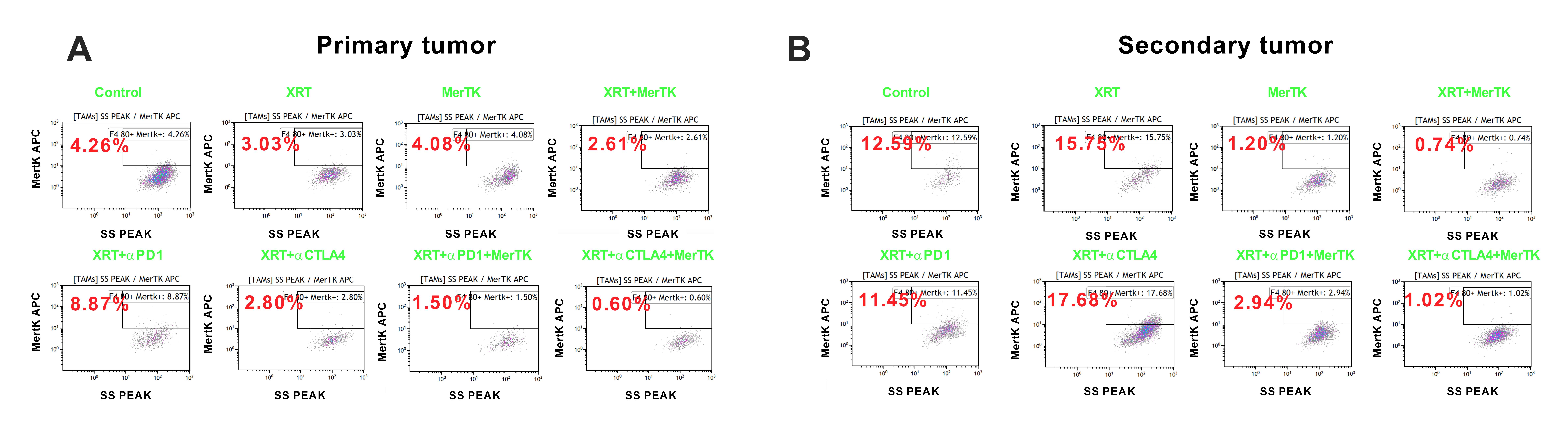

Supplement: Supplementary file 3 — Additional file 3: Supplental Fig. S3. Representative FACS images of MerTK+ macrophages. A, Primary tumors. B, Secondary tumors. [file 13046_2024_2992_MOESM3_ESM.jpg]

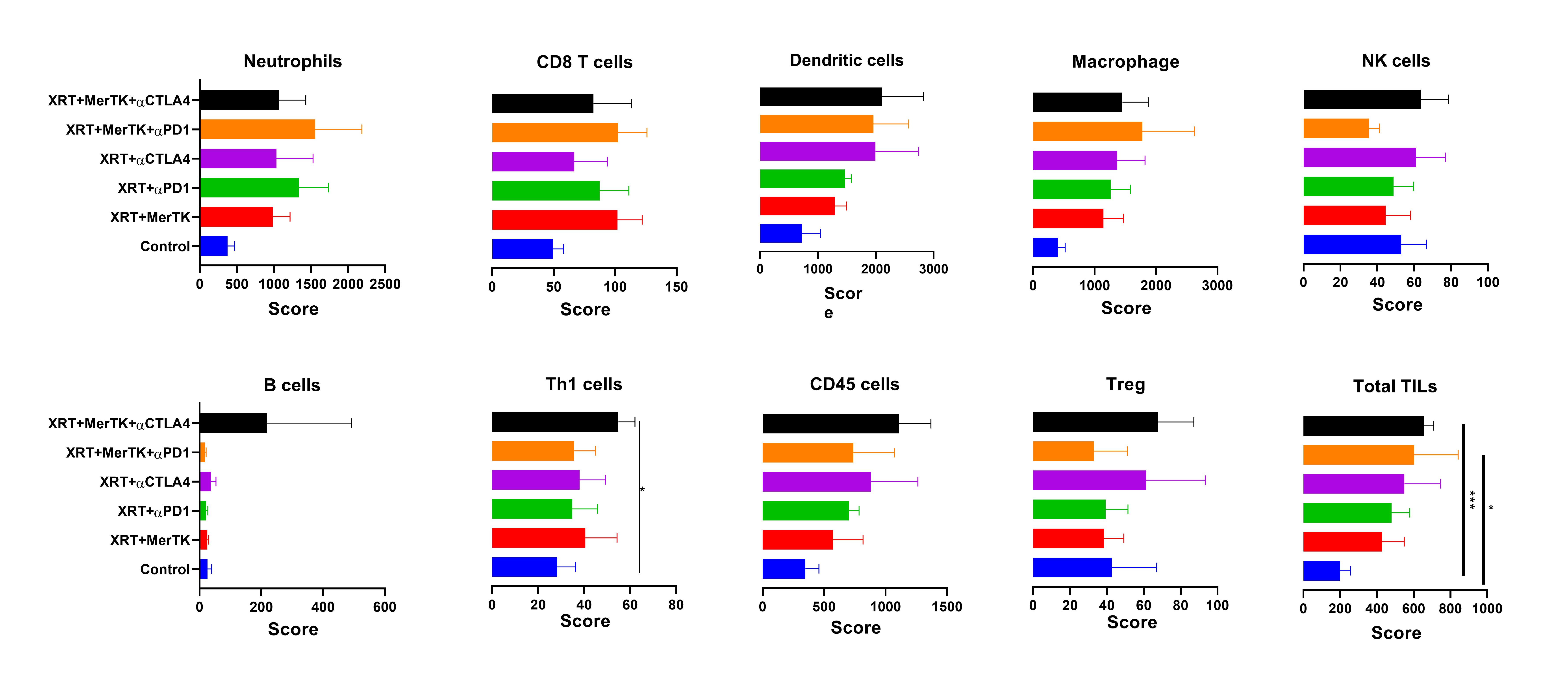

Supplement: Supplementary file 5 — Additional file 5: Supplemental Fig. S5. NanoString scores of various immune cells in the primary tumors. The mice (n=3) were treated with different combinations of XRT, MerTK ASO, anti-PD1, and anti-CTLA4 as indicated in Fig. 1A and Fig. 2A and were sacrificed on day 16. The total RNA extracted from the primary tumors was analyzed with a nCounter PanCancer Immune Profiling Panel. All the statistics were expressed as mean value ± SEM. [file 13046_2024_2992_MOESM5_ESM.jpg]

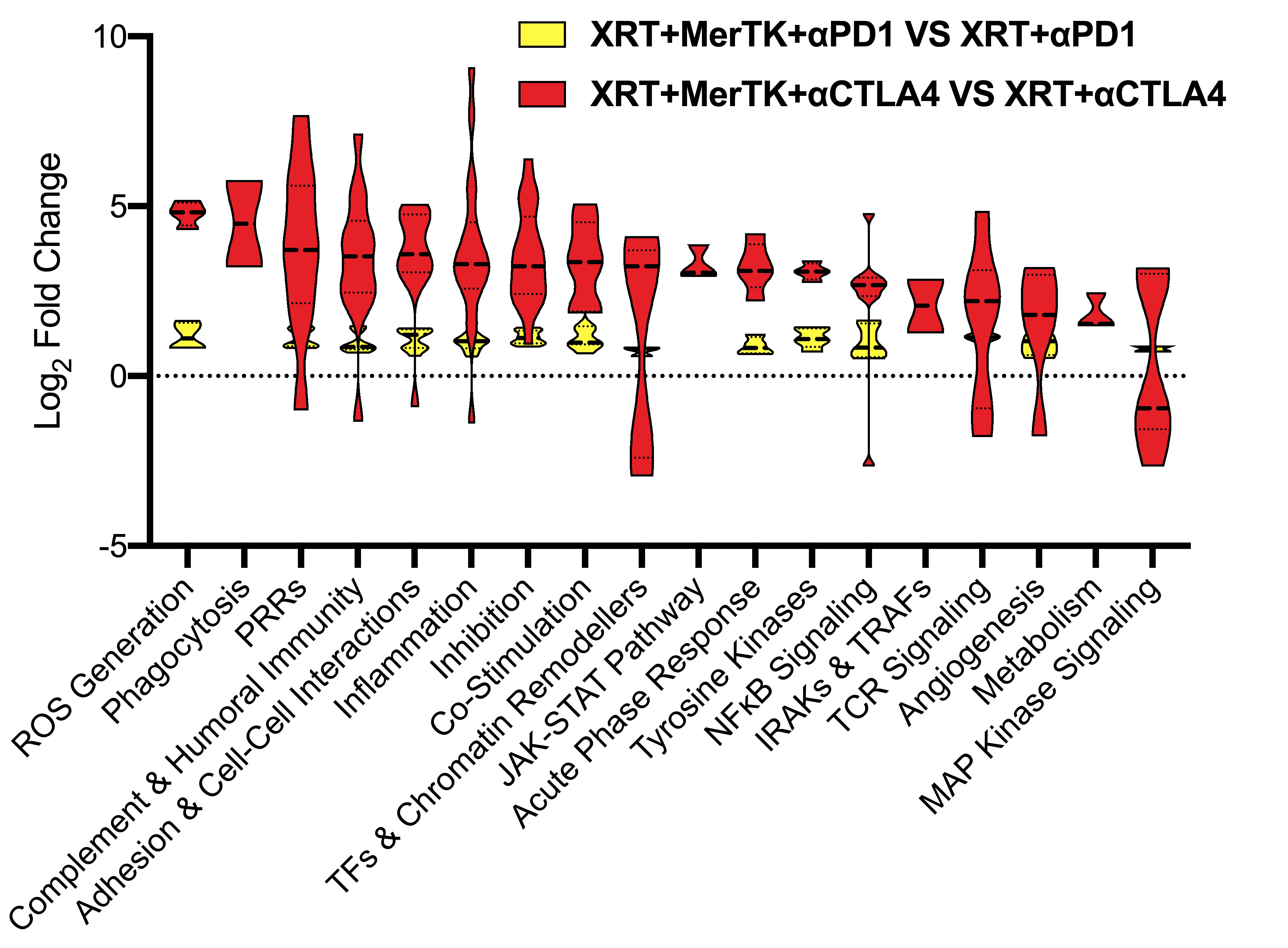

Supplement: Supplementary file 7 — Additional file 7: Supplemental Fig. S7. MerTK ASO significantly changed the activity of various pathways in the secondary tumor when added to XRT+aPD1 and XRT+aCTLA4. [file 13046_2024_2992_MOESM7_ESM.jpg]

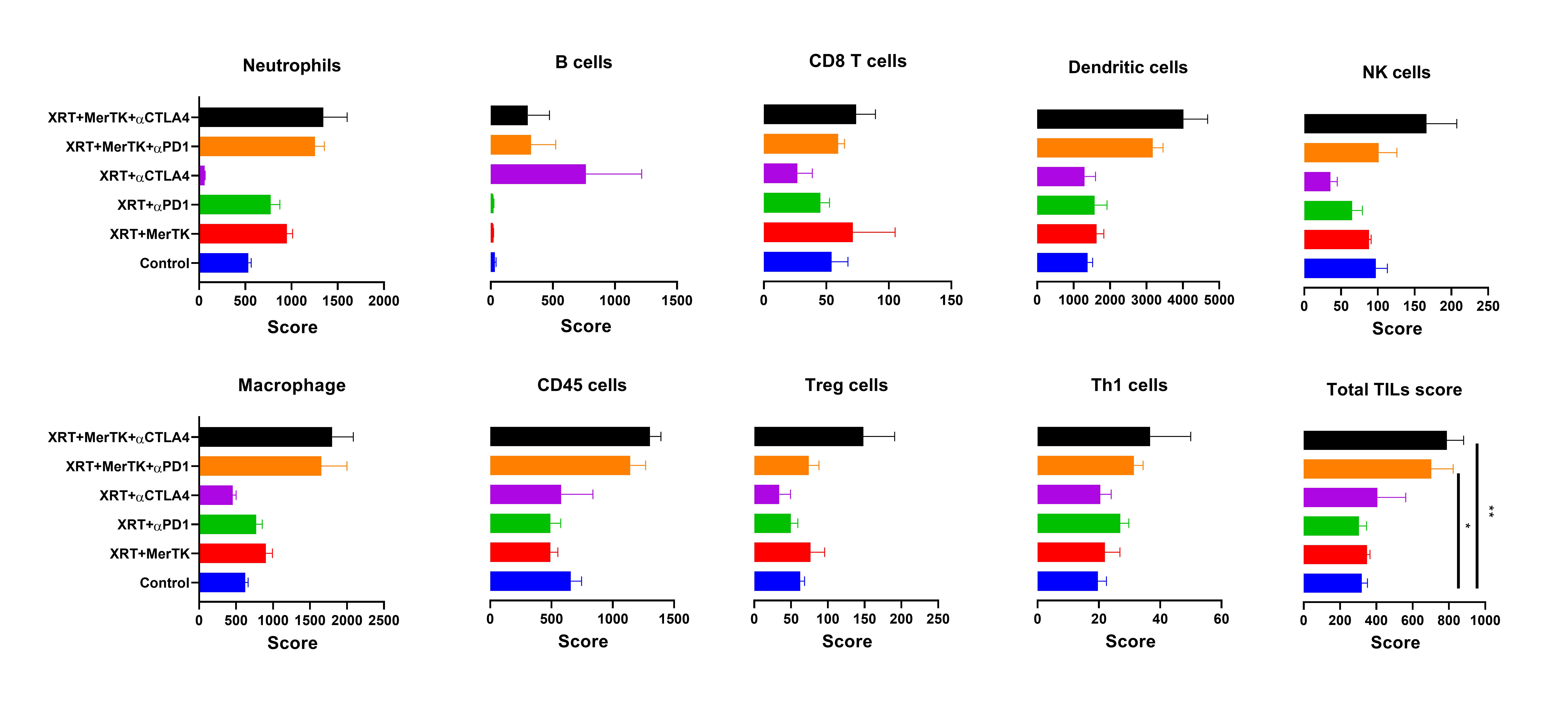

Supplement: Supplementary file 8 — Additional file 8: Supplemental Fig. S8. Nanostring scores of various immune cells in the secondary tumors. The mice (n=3) were treated with different combinations of XRT, MerTK ASO, anti-PD1, and anti-CTLA4 as indicated in Fig. 1A and Fig. 2A and were sacrificed on day 16. The total RNA extracted from the primary tumors was analyzed with an nCounter PanCancer Immune Profiling Panel. All statistics were expressed as mean value ± SEM. [file 13046_2024_2992_MOESM8_ESM.jpg]

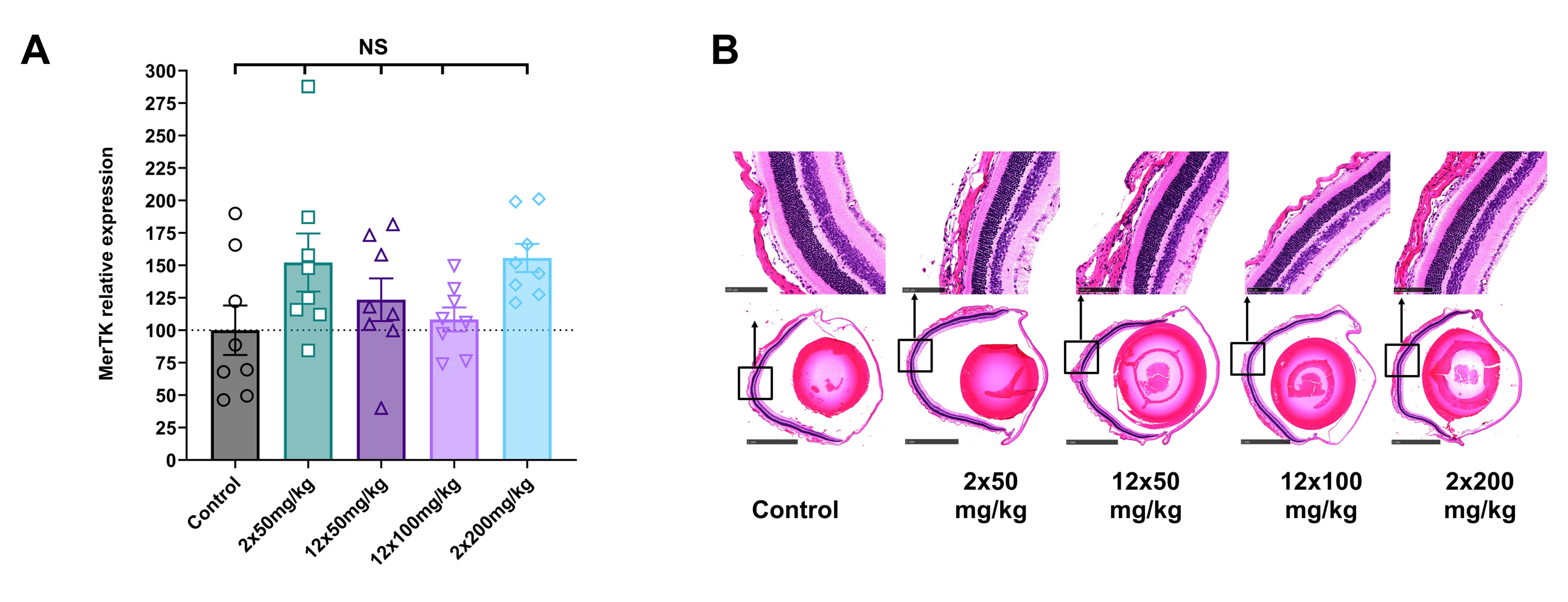

Supplement: Supplementary file 9 — Additional file 9: Supplemental Fig. S9. Evaluation of dose-dependent effects of MerTK ASO on MerTK expression and ocular pathology. Female 129Sv/Ev mice aged 8-12 weeks (n=8 per group) received treatments with varying dosages of MerTK ASO: 50 mg/kg on days 8 and 12 (total 2x50 mg/kg), 50 mg/kg on days 1-5, 8-12, 15, and 19, 100 mg/kg on days 1-5, 8-12, 15, and 19, and 200 mg/kg on days 8 and 12. On day 22, the mice were euthanized, and their eyes were excised. MerTK expression was analyzed via RT-PCR. Additionally, the eyes underwent Hematoxylin & Eosin (H&E) staining to assess potential pathological alterations. [file 13046_2024_2992_MOESM9_ESM.jpg]
